# Ecology, evolution, and management strategies of northern pike populations in the Baltic Sea

**DOI:** 10.1007/s13280-015-0664-6

**Published:** 2015-05-28

**Authors:** Per Larsson, Petter Tibblin, Per Koch-Schmidt, Olof Engstedt, Jonas Nilsson, Oscar Nordahl, Anders Forsman

**Affiliations:** Institute of Biology and Environmental Science, Linnaeus University, 391 82 Kalmar, Sweden

**Keywords:** Climate change, Conservation, *Esox lucius*, Habitat restoration, Homing, Population divergence

## Abstract

Baltic Sea populations of the northern pike (*Esox lucius*) have declined since the 1990s, and they face additional challenges due to ongoing climate change. Pike in the Baltic Sea spawn either in coastal bays or in freshwater streams and wetlands. Pike recruited in freshwater have been found to make up about 50 % of coastal pike stocks and to show natal homing, thus limiting gene flow among closely located spawning sites. Due to natal homing, sub-populations appear to be locally adapted to their freshwater recruitment environments. Management actions should therefore not involve mixing of individuals originating from different sub-populations. We offer two suggestions complying with this advice: (i) productivity of extant freshwater spawning populations can be boosted by modifying wetlands such that they promote spawning and recruitment; and (ii) new sub-populations that spawn in brackish water can potentially be created by transferring fry and imprinting them on seemingly suitable spawning environments.

## Introduction

The Baltic Sea is one of the largest brackish water areas on Earth with a unique ecosystem where species of both freshwater and marine origin have been established (Wennerström et al. 2013). However, since the 1960s, there has been a continuous deterioration of the Baltic Sea ecosystem due to anthropogenic activities as overfishing, eutrophication, coastal exploitation, and global climate change (Elmgren 2001). This has resulted in large-scale trophic cascades with significant impacts on the ecosystem function. Several predatory fish species, for example, northern pike (*Esox lucius*) and cod (*Gadus morhua*), have declined, whereas zooplanktivorous fish species, in particular three-spined stickleback (*Gasterosteus aculeatus*) and sprat (*Sprattus sprattus*), have increased (e.g., Nilsson et al. 2004; Casini et al. 2008). As a consequence, abundances of zooplankton and invertebrates have decreased, whereas phytoplankton and filamentous algae have increased (Eriksson et al. 2009; Ljunggren et al. 2010; Sieben et al. 2011).

The coastal fish community of the Baltic Sea is dominated by species of freshwater origin, e.g., northern pike, perch (*Perca fluviatilis*), roach (*Rutilus rutilus*), and three-spined stickleback, although species of marine origin, as cod, herring (*Clupea harengus membras*), and sprat, are also present. Some species of freshwater origin (Table 1), for example, coastal keystone species as northern pike, pikeperch (*Sander lucioperca*), and perch, have anadromous life-history strategies similar to those of salmonids; they use the coastal environment in the Baltic Sea as forage habitat but migrate to streams and brooks for reproduction (Müller and Berg 1982; Engstedt et al. 2010; Tibblin et al. 2012; Engstedt et al. 2014; Rohtla et al. 2014). Those species have declined since the 1990s, and eutrophication, habitat loss (e.g., exploitation of coastal bays and ditching of adjacent wetlands and streams), overfishing, and trophic cascades have been proposed as possible causes (Nilsson et al. 2004; Nilsson 2006; Eriksson et al. 2009; Lehtonen et al. 2009; Ljunggren et al. 2010; Sieben et al. 2011; Mustamäki et al. 2014; Sundblad et al. 2014).

**Table 1:** Fish species in the Baltic Sea that have been documented to undertake spawning migrations to freshwater or species that spawn both in freshwater and in the brackish environment

Species		Reference
Burbot	*Lota lota*	Müller and Berg (1982) and Rohtla et al. (2014)
Crucian carp	*Carassius carassius*	Müller and Berg (1982)
Dace	*Leuciscus leuciscus*	Müller and Berg (1982)
European smelt	*Osmerus eperlanus*	Lajus et al. (2007)
Grayling	*Thymallus thymallus*	Müller and Berg (1982)
Ide	*Leuciscus idus*	Müller and Berg (1982)
Northern pike	*Esox lucius*	Müller and Berg (1982) and Engstedt (2011)
Perch	*Perca fluviatilis*	Müller and Berg (1982)
Pikeperch	*Sander lucioperca*	Lappalainen et al. (2003) and references therein
River lamprey	*Lampetra fluviatilis*	Müller and Berg (1982), Thiel et al. (2009) and references therein
Roach	*Rutilus rutilus*	Müller and Berg (1982)
Ruffe	*Gymnocephalus cernuus*	Müller and Berg (1982)
Sea lamprey	*Petromyzon marinus*	Thiel et al. (2009) and references therein
Sturgeon (extinct)	*Acipenser* ssp.	Lajus et al. (2007)
Vimba bream	*Vimba vimba*	Lajus et al. (2007)
Whitefish	*Coregonus* spp.	Aronsuu and Huhmarniemi (2004) and Lajus et al. (2007)

The coastal fish community in the Baltic Sea is challenged also by environmental changes associated with climate change. Over the next hundred years, scenarios for the Baltic Sea area forecast a few degrees higher temperature and a substantial increase (~30 %) in precipitation especially in the northern part of Baltic Sea drainage area. Climate change models further project milder winters with less ice cover, and that the low-salinity gradient in surface water will expand southwards (Neumann 2010; Wake 2012). It is difficult to foresee how these more long-term changes will influence coastal fish populations. However, in a shorter perspective, overfishing and habitat modifications pose severe threats to many species of fish (Österblom et al. 2007; Sundblad and Bergström 2014). This calls for continued research, development of management plans, and implementation of practical restoration actions to boost recruitment and population growth of top-predator fish.

The spawning and recruitment areas for migrating fish can be improved by small means. Opening of migration routes, restoration of wetlands, or construction of spawning and recruitment habitats may improve coastal stocks (Nilsson et al. 2014). However, different fish species have specific demands regarding spawning and recruitment areas. Ecological knowledge of the species is therefore crucial for successful outcomes. For several species, current knowledge is based only on studies in freshwater environments, and this is not necessarily applicable to coastal areas.

Here, we examine the ecology of the northern pike and offer examples of ways by which the coastal stocks can be improved. We discuss caveats that must be taken into consideration when developing management plans for improved recruitment and more viable coastal stocks. Further, we show how different approaches can be used to reveal key ecological and evolutionary processes. Finally, we discuss how climate change may influence coastal fish populations in the Baltic Sea.

## Northern pike

Northern pike is a large (<130 cm) and long-lived (<20 years) keystone predatory fish that is emerging as an important model organism for studies of ecology and evolution (Forsman et al. 2015). Pike can influence fish communities, shaping composition as well as abundance and distribution of their prey, and this may also effect other trophic levels (Craig 1996). The decline of pike stocks along the Swedish Baltic coast is postulated to have caused cascading ecosystem effects in coastal fish and vegetation communities (Ådjers et al. 2006; Ljunggren et al. 2010; Sieben et al. 2011).

Sympatric populations of pike in the Baltic Sea have two different reproductive strategies (Engstedt et al. 2010). They either spawn in brackish coastal waters or in freshwater streams with adjacent wetlands and small lakes but share a common coastal habitat during the majority of their life cycle. The anadromous populations start their spawning migration in early spring, and spawning takes place in shallow vegetated areas (Müller 1986; Nilsson 2006; Engstedt 2011). Areas of flooded vegetation offer good spawning conditions and food resources for the fry and provide refuge from predation (Lappalainen et al. 2008; Nilsson et al. 2014). The larvae display a clear distribution pattern after hatching preferring shallow vegetated areas. Most juveniles stay less than one month (at a size <6 cm) in freshwater before emigrating to the Baltic Sea (Nilsson et al. 2014). This early emigration may be a way to cope with seasonally decreasing water levels and avoid cannibalism.

## Spawning migrations and sampling of fish

Over the years, there have been several observations that fish species such as pike, perch, ide (*Leuciscus idus*), and whitefish (*Coregonus* spp.) undertake seasonal migrations from coastal areas in the Baltic Sea to adjacent freshwater streams (Müller and Berg 1982; Karås and Lehtonen 1993). The aggregations in watercourses are extensive, and one reason for this behavior is spawning.

We studied the migration of pike in six small neighboring streams at the southwest Baltic Sea coast (56°40′N, 16°20′E). Because streams were adjacent any of the migrating fish could potentially reach any of the streams to spawn, and that environmental variables possibly affecting migration behavior of adults were the same. Moreover, the low water flows during summer prevent establishment of resident pike; pike only resides in the small streams during reproduction and larval period (Engstedt 2011). Working in small streams also enables better control of fish sampling over years.

**Fig. 1:**
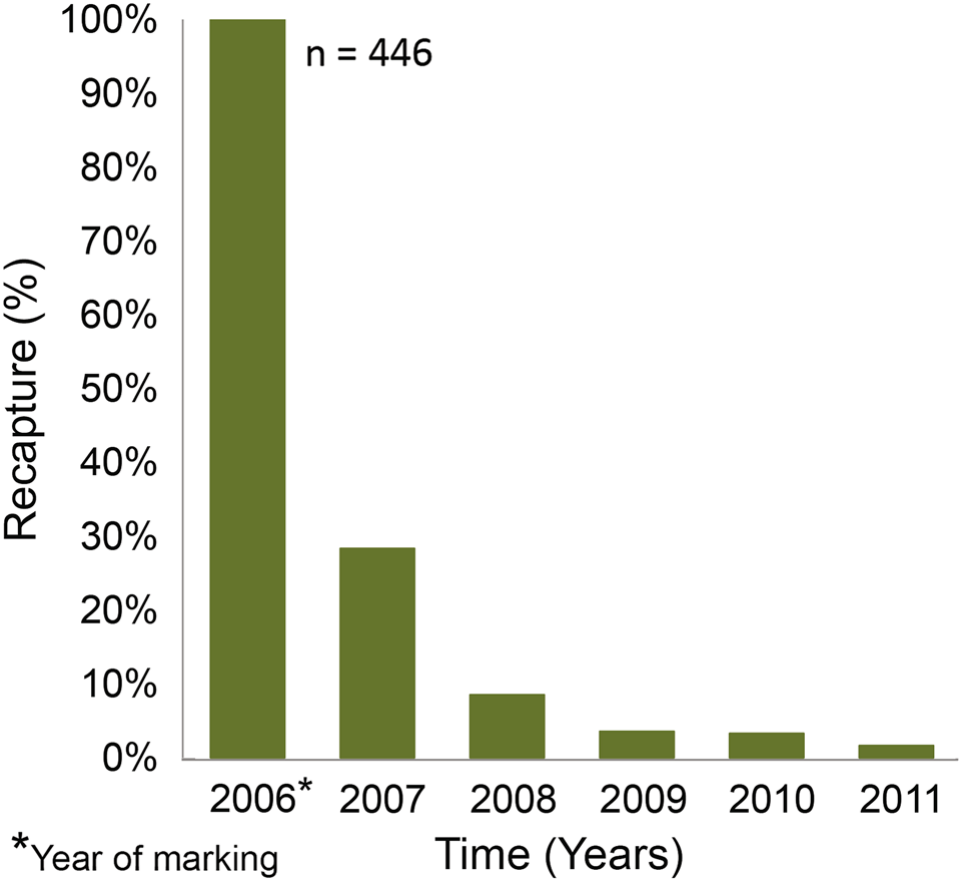
Pike marked in a brook/wetland (Lervik) during the first spawning migration year and returns the following year(s)

We marked >3000 mature migrating pike (weighing 0.5–11.5 kg) in the streams from 2005 to 2010. Fish were caught using stream-wide fyke nets placed approximately 100 m from where the streams entered the sea. Fish were permanently marked with electronic passive integrated transponder (PIT) tags inserted into the body cavity. PIT-tag stations recording “tag” passes were placed at one or several sites in each stream and were activated for either a whole year or during the spawning migration in spring. Fish were sexed, measured for body length, and weighed, and a tissue sample was taken for genetic analysis. Some individuals were sacrificed and sampled for otoliths and cleithra used for analyses of trace elements to determine place of origin, temporal variation in habitat use, and to reconstruct past growth rates. Return rates of migrating pike the first year after marking averaged 36 % (22–45 %) in the spawning streams and decreased to 22 % of the originally marked fish in the second year and to 15 % in the third year (Fig. 1) (Engstedt 2011). The high proportions of pike returning to the spawning grounds year after year indicate homing, and the spawning stream fidelity was high. No electronically tagged fish had gaps in their spawning recapture history (e.g., returning after two consecutive years at sea), and no fish was recaptured in a stream different from where it was originally marked and released.

## Otoliths can inform about origin, age, growth, movements, and habitat use

**Fig. 2:**
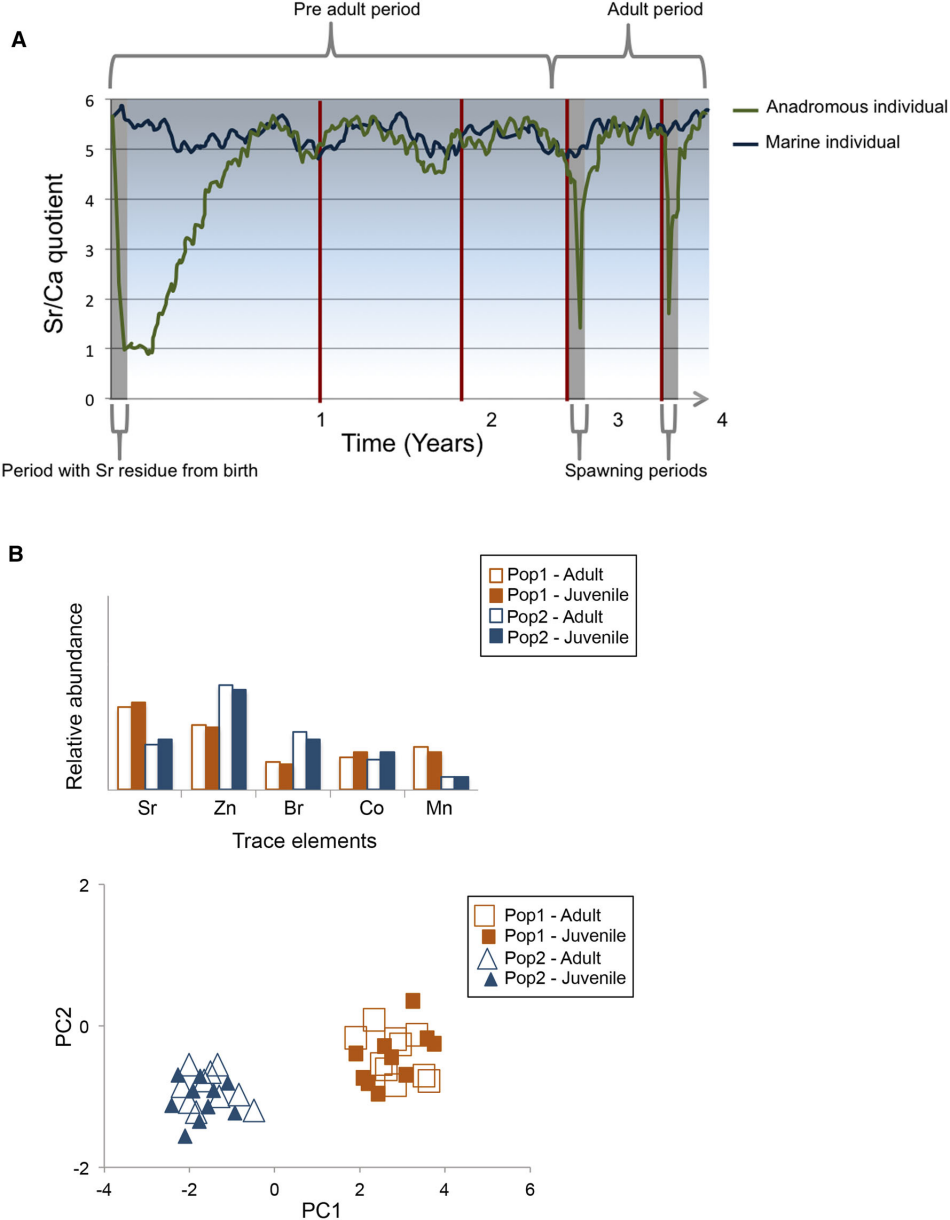
The Sr/Ca signal in an otolith from a pike born and spending the juvenile phase in a freshwater habitat and an individual spending the whole life in the sea (**a**). The “mother” signal in the core of the otolith origins from the anadromous mother transferring Sr to the egg during her marine foraging (*left*). The unique element pattern (**b**, element fingerprint) in otoliths from juvenile pike in different brooks/wetlands and from mature pike returning to their birth brook (their former juvenile phase). Principal drawings modified after Engstedt et al. (2014)

Otoliths are mainly composed of calcium carbonate (CaCO_3_) and part of the hearing and balance system in teleost fishes. They grow with the fish as new layers of CaCO_3_ are deposited on the surface, creating year rings that reflect periods of growth and starvation (i.e., during winter at latitudes covering the Baltic Sea). Otoliths can consequently be used for age determination and to reconstruct past growth trajectories. The resolution of “time” in the otoliths is coupled to the relative growth rate of the fish; a year in the beginning of the fish life (from the core to the first year ring) covers a larger distance than a year later in life (Fig. 2a). Otoliths also contain trace elements taken up from water and may reveal present or passed habitats of the fish (Campana 1999; Walther and Limburg 2012). The process is especially pronounced in fish that migrate between freshwater and marine environments (Walther and Limburg 2012). A useful element indicator of this type of migration is strontium (Sr), which accumulates at a higher rate in the otoliths when the fish is in the sea, due to higher concentrations, and decreases if the fish migrates to freshwater habitats. We examined the otoliths of adult pike caught in the southwest part of the coastal Baltic Sea and found that 46 % were born in freshwater, and that the rest were of marine origin (Engstedt et al. 2010). This shows that a significant fraction of the pike in this part of the Baltic Sea originates from freshwater spawning and recruitment areas.

Experimental studies has confirmed that Sr measurements in otoliths of pike can inform about habitat use (Engstedt et al. 2012). Uptake of Sr in the otoliths was directly proportional to the salinity in the Baltic Sea water. Consequently, Sr concentrations could be used to reconstruct the salinity of habitats used by pike during their lifetime. Anadromous pike showed a consistent pattern, with a juvenile freshwater phase followed by a foraging period in the sea, and with a cyclical post maturity phase characterized by yearly shifts between short periods in freshwater environments during spawning and longer periods in the sea for foraging and growth (Fig. 2a). Pike born in the sea showed no patterns of oscillating Sr concentrations in the otoliths.

Trace elements other than Sr are also taken up in the otolith, and the element composition (fingerprint) reflects the history of habitat use (Engstedt et al. 2014). This allows for reconstruction of movement patterns at a finer spatial scale than between fresh and brackish water environments. We examined the element fingerprints (Sr, Zn, Br, Co, and Mn) in juvenile pike that originated from different streams entering the Baltic Sea. We found a unique element composition pattern in the juveniles from different streams, showing that water chemistry in the aquatic habitats was reflected in the otoliths, thus revealing birth origin and juvenile growth. Next, we caught migrating adults in the spawning streams. Using the Sr signal in the otoliths as a freshwater reference (from the core (birth) to the higher Sr signal indicating that migration to the sea was completed), the element fingerprints during the juvenile period could be defined for adult fish (Fig. 2b). Juvenile and adult fingerprints were similar within streams, indicating that the adult fish were born in the same spawning area as the juvenile. This match in trace element composition was evident despite that the juvenile period of adult fish had occurred several years before the juvenile fish were caught (Fig. 2b) (Engstedt et al. 2014). The results thus demonstrate natal fidelity (homing); the adult pike return to spawn where they were born.

## Causes and consequences of natal homing and spawning site fidelity

The ecology and evolution of an anadromous lifecycle that includes homing behavior has been thoroughly examined in salmonids, in particular in Atlantic salmon (Gross 1991). A few studies have examined spawning site fidelity in Esocidae fishes, including northern pike (Miller et al. 2001) and muskellunge (*Esox masquinongy*) (Crossman 1990), and provide some indications of natal homing. Previous studies have used either the ‘capture mark recapture’ method in spawning grounds or genetic markers. The ultimate reasons for homing behavior in pike may be the same as for salmon. Food resources in the Baltic Sea are high, and this enables the emigrating fry to grow fast (Gross 1987; McDowall 1997), and as fish become larger, their reproductive capacity and fecundity increase. The nursery areas in wetlands and flooded areas connected to freshwater streams offer protection against predation on juveniles, which is intense in the sea (Nilsson 2006; Nilsson et al., unpubl. results). Smaller individuals of a fish species are known to have a higher risk of mortality, and migrating fish are especially at risk (Jonsson and Jonsson 2004). However, the high density of pike in the streams before, during, and after spawning also renders slow growing juveniles at greater risk of cannibalism in the nursery areas (Skov et al. 2003; Persson et al. 2004). On the other hand, wetlands warm quickly in early spring when the fry hatch, and nutrients accumulated in winter promote development of zooplankton that pike feed on during early life stages (Nilsson et al. 2014).

**Fig. 3:**
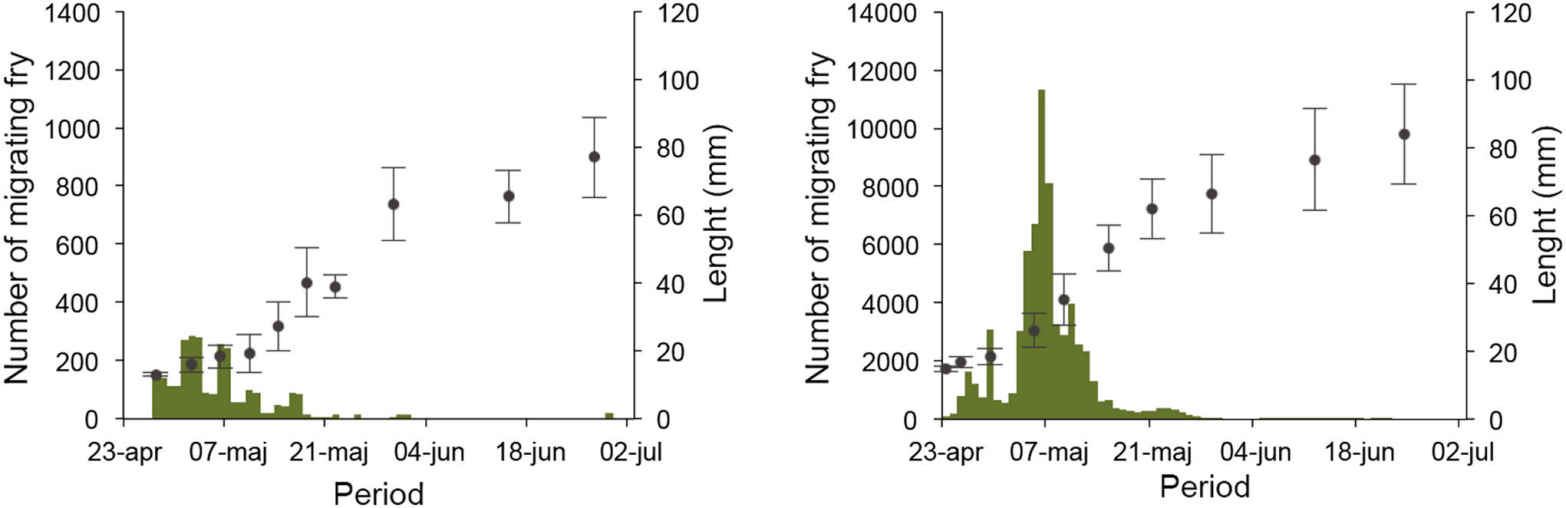
Fry migration to the sea over a season (*left axis*) and the growth (*right axis*) from the Oknebäck wetland before and after restoration. Redrawn from Nilsson et al. (2014)

We examined emigration of fry from one of the streams by trapping individuals over three spawning seasons and found that production in one season was over 30 000 fry per hectare (Fig. 3) (Nilsson et al. 2014). This bears testimony of the high productivity and indicates that recruits stemming from spawning in freshwater environments may contribute substantially to the total population of pike in coastal areas. However, natal homing and spawning site fidelity also have important implications for population genetic structure and evolutionary divergence among sub-populations.

## Homing contributes to population structure and allows for local adaptations

**Fig. 4:**
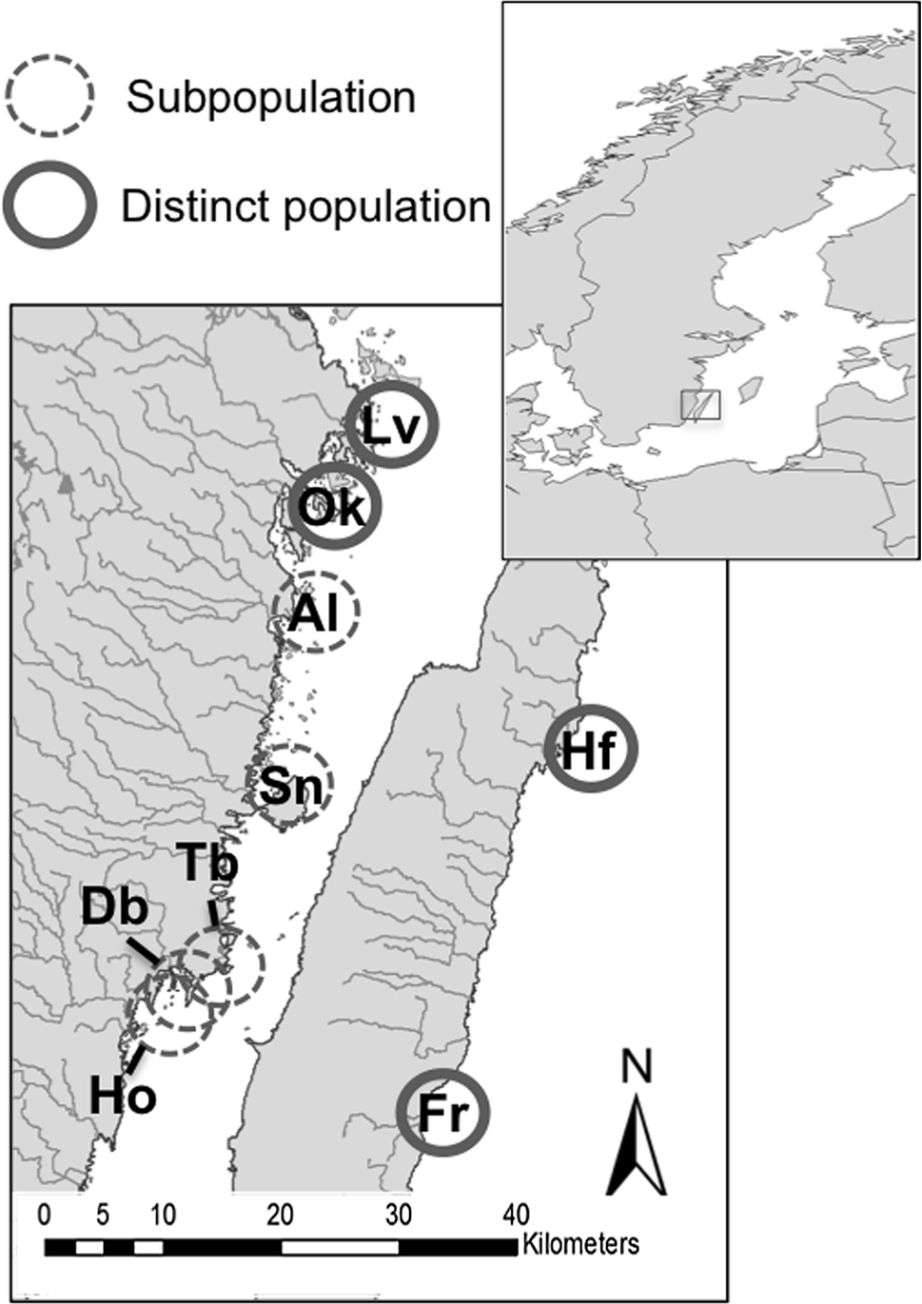
Microsatellite analysis of juvenile and mature pike caught in different freshwater habitats (recruitment and spawning habitats). The analysis reveals (sub) population development induced by spawning barriers (homing). Subpopulation (Mean Pairwise *F*_st_ Range 0.01–0.05; *p* < 0.05). Distinct population (Mean Pairwise *F*_st_ Range >0.05; *p* < 0.05)

We examined the genetic relationships of pike spawning in different streams. Analyses of ten nuclear microsatellite DNA loci (Miller et al. 2001; Jacobsen et al. 2005; Laikre et al. 2005) of approximately 300 northern pike indicated genetic differences between fish from different spawning streams, as well as genetic similarities among juveniles and migrating adults from the same stream and over time (Fig. 4). These results cannot be attributed only to isolation by distance, because spawning streams were close to each other and could have been reached by any fish in the coastal area. More likely, the observed genetic structure has arisen because adult pike repeatedly return to spawn in the same stream where they were born. This conclusion is supported by the finding in the marking study that no fish was recorded in a stream different from where it was originally marked. We conclude that pike exhibits natal homing and spawning site fidelity, and that this has resulted in partial reproductive isolation and evolution of genetically differentiated sub-populations.

**Fig. 5:**
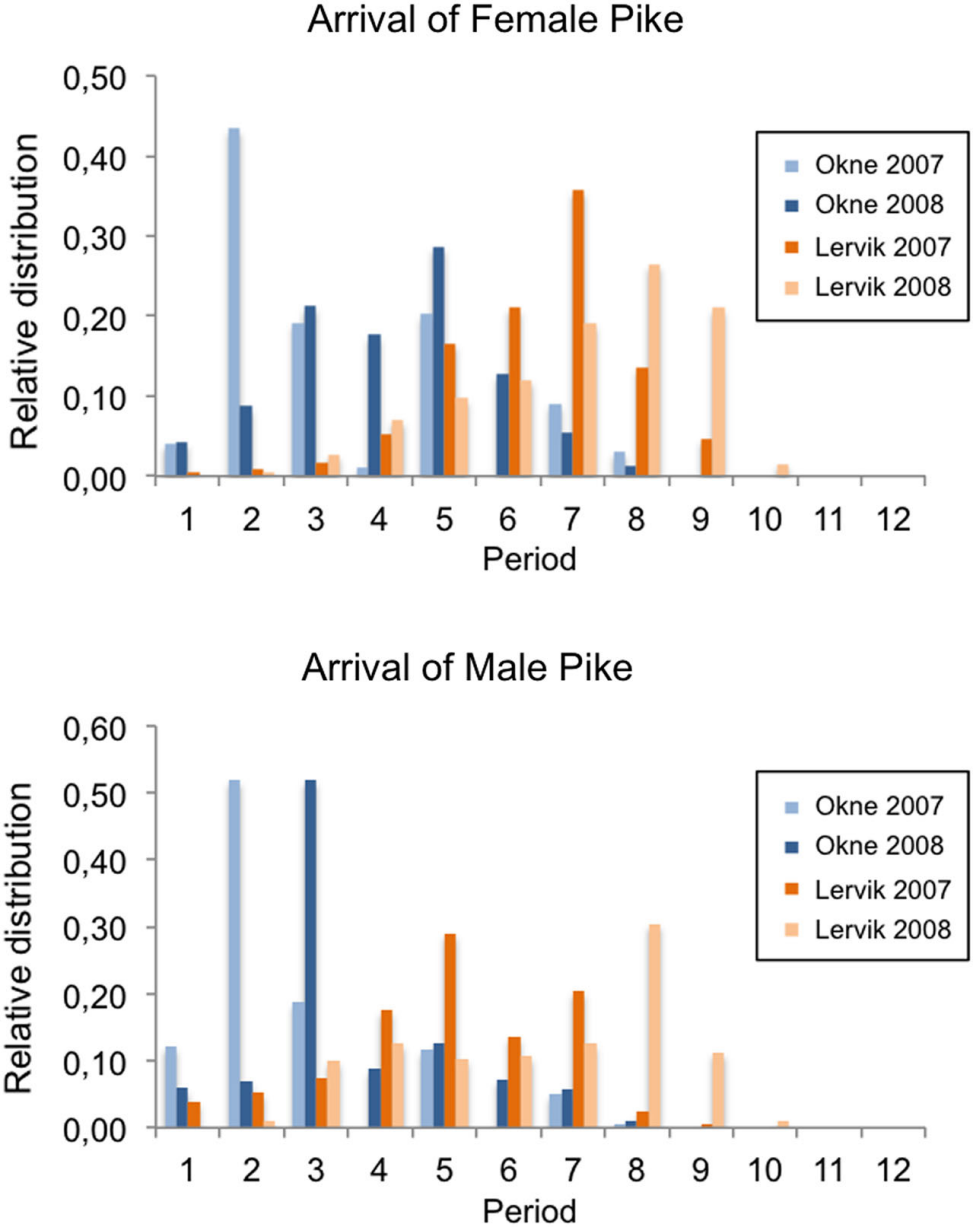
Frequency of adult pike migration over the season to two nearby freshwater spawning brooks/wetlands. Individuals of one population arrive earlier for spawning than the other. The difference in timing is most pronounced for females, males generally arrive earlier due to pike spawning behavior. Period 1 (first registration of individual fish) starts on March 17, and each period covers 4 days. *Upper* Spawning migration of female pike in 2007 and 2008. The distribution is based on 570 fishes in Oknebäck and 618 fishes in Lervik. *Lower* Spawning migration of male pike in 2007 and 2008. The distribution is based on 570 fishes in Oknebäck and 700 fishes in Lervik

Fish from different sub-populations differ not only in neutral genetic markers, but they also show signs of evolutionary divergence in phenotypic traits, possibly reflecting local adaptations of life-history characteristics such as timing of reproduction, reproductive allocation, growth rate, and body size (Nordahl 2013; Tibblin et al. 2015). For instance, we compared temporal migration patterns of pike in two nearby streams (Fig. 5). Male pike generally arrive to the spawning areas earlier than females (Frost and Kipling 1967). However, both females and males arrived earlier (about 20 days) to one of the spawning areas, and this pattern was consistent over the two common years examined. The difference in reproductive timing between these two populations may represent a response to seasonal water discharge and retention time, because survival of fry is dependent on free migration routes to sea.

Data on reproductive investment in female pike collected from different streams during spawning have uncovered differences in reproductive effort, fecundity, and egg size, suggesting that the sub-populations have their own suites of life-history characteristics (Nordahl 2013). This conclusion was further corroborated when we examined growth rates of juvenile pike in a laboratory common garden experiment (Tibblin et al. 2015). Moreover, results from a reciprocal translocation experiment show signs of local adaptation (Berggren et al., unpubl. results). This example of evolutionary divergence is remarkable, because individuals are physically separated for only a short period during the earliest larval and juvenile stage, while using a common coastal environment for foraging and growth during most of their life.

## Consequences of population subdivision and local adaptations for management and conservation of coastal fish stocks

The natal homing, spawning site fidelity, and resulting genetic structuring into sub-populations of pike of freshwater origin in the Baltic Sea have important implications for fishery management of coastal stocks. If pike are to be stocked in coastal areas, practitioner managers need to consider using parental fish that originate from the specific area. Otherwise, the genetic lineages might be mixed, and adaptations for spawning in freshwater versus brackish environments can be lost (Gilk et al. 2004; McClelland and Naish 2007a). Additionally, measures can be taken to improve spawning and recruitment areas in watercourses entering the Baltic Sea and thereby strengthen coastal stocks of pike and perch. By improving spawning and recruitment areas already utilized by adult fish such that natural populations become more productive, the genetic architecture is not changed, as would be the case if fish of unknown or mixed origin raised under captive conditions were used for stocking. If fish from the specific population are used as the base for recruitment, adaptations important for survival and reproduction within a certain coastal area can be maintained. If farmed fish are to be used for stocking, the origin of the fish needs to be considered. It is often recommended that one should primarily use breeding-stocks from the same region or general area to which the progeny are to be released. However, our studies of pike demonstrate that geographic proximity cannot be used as a reliable proxy for genetic and ecological similarity.

## Restoration of wetlands may increase recruitment of freshwater spawning pike populations

Since the 1990s, pike abundance has decreased in many coastal areas throughout the Baltic Sea (Laikre et al. 2005; Nilsson 2006; Lehtonen et al. 2009; Ljunggren et al. 2010). The coexistence of migrating, freshwater-born, and sea-born pike in the Baltic could reflects a balance between, on one hand, the risk of mortality during migration *versus* more surviving fry and, on the other, the lower risk of mortality for mature sea-dwelling fish *versus* fewer surviving fry. Fish with an anadromous life cycle are sensitive to changes in their freshwater spawning and recruitment areas, and pike would be affected by changes associated with ditching and construction of dams. For pike of sea origin, eutrophication and the degradation of coastal reproduction habitats may negatively affect recruitment (Sundblad and Bergström 2014). One way of counteracting the decrease in pike in parts of its former distribution range along the coast is to improve or restore wetlands and flooding areas in tributaries (Nilsson et al. 2014). Many wetlands have been restored and new ones constructed to reduce nutrient emissions to the sea, and small measures such as constructing fish passes and opening waterways may contribute to improved recruitment areas for migrating pike. Optimal conditions for denitrification (Fisher and Acreman 2004) and phosphorous catchment, such as high temperature, shallow water areas with diverse and patchy vegetation, also create good microhabitats for pike spawning and fry recruitment (Box 1). Such wetland restoration measures are under development in Sweden (Nilsson et al. 2014).

**Box 1:** Criteria for optimal spawning- and nursery habitats in wetlands for pike (*Esox lucius*)

*Vegetation*
Temporarily flooded terrestrial vegetation (hummocks of grasses and sedges), fragmented and moderately dense creating microhabitats. Aquatic vegetation (reed and mosses etc.)
*Water depth*
A depth of 10–70 cm, mean 20–50 cm. Gradually increasing to spawning. Stable or slowly decreasing until the fry migrate (within two months after spawning)
*Exposure*
Sheltered, warming rapidly in early spring, receiving direct sunlight from south or west
*Availability*
Small open waterways (fish passes) into (and out of) the wetland for spawning fish and emigrating fry
*Substrate*
Plant detritus well oxygenated. Good medium for rooting of grasses and sedges. No areas with free sediment than can be suspended
*Water exchange*
“Medium”, not too fast to counteract the heating effect

Modified after Casselman and Lewis (1996) and Nilsson et al. (2014)

When we examined the emigration of fry from one of the restored wetlands (Nilsson et al. 2014), we found that total annual emigration increased from about 3000 juveniles before restoration to >100 000 the year after restoration, and the effect was maintained the coming year. Five years after the restoration, emigration was >300 000.

## Projecting and safeguarding against consequences of climate change

The consequences of climate change and associated environmental shifts for species and populations are simple in principle: they can respond by range shifts; shifts in the seasonal timing of reproductive activities, adaptation via evolutionary (genetically based) change, or phenotypic plasticity; or become extinct. How will climate change influence pike and other fish species in the Baltic Sea? A prognosticated 30 % increase in precipitation will drive the freshwater gradient to the south, thereby expanding low-salinity coastal habitats (Neumann 2010; Wake 2012). This may be beneficial for species of freshwater origin that spawn in coastal areas. A spatial and temporal decrease in Baltic Sea ice cover will prolong the foraging season for most fishes. Climate change may also affect the hydrological regimes in coastal freshwater streams (Graham 2004). Models point to a higher winter discharge as a result of increased winter precipitation and lower spring and summer discharge due to the reduced winter snow storage and an increase of evapotranspiration (Neumann 2010; Wake 2012). As a consequence, potential spawning areas may fill up and flood earlier than before, such that recruitment areas suitable for fry may become larger and more abundant. On the other hand, wetlands may be drained more quickly, as the timing of peak flows will occur earlier in season. Environmental changes associated with continued global warming discussed above may thus have both positive and negative effects for both freshwater and marine spawning sub-populations of pike in the Baltic Sea. Given this uncertainty, our advice is to adopt a bet-hedging management strategy, and that resources and actions are directed in ways that promote the productivity of both the freshwater and the brackish water spawners.

Not much is known about the ecology and dynamics of pike that spawn in coastal areas of the Baltic Sea, and there is a need for more research to fill this gap. Further studies of the brackish water populations are called for also because the decline of pike stocks in the Baltic Sea has been pronounced along the coast (Nilsson et al. 2004; Ljunggren et al. 2010). In the wait for more knowledge and better informed management plans, one can monopolize on the natal homing behavior of pike as a means to re-introduce spawning populations. In view of their homing behavior, it seems that fry may become imprinted on their birth area and return after maturity to the same area for spawning. If so, pike fry could be transferred to previously used, but currently deserted, spawning areas along the coastline and in outer archipelagos, to founder additional brackish water sub-populations and strengthen coastal fish stocks. Measures should be taken to prevent further deterioration of spawning habitats and to fishing pressure (Sundblad and Bergström 2014; Sundblad et al. 2014). This might promote natural re-establishment of pike in deserted areas (Pukk et al. 2013). Successful management of anadromous populations of northern pike may counteract eutrophification effects by top-down control of underlying trophic levels. This will likely have a positive impact on the coastal Baltic ecosystem in general, including northern pike and other predatory fish spawning in brackish water.

It remains uncertain how the effects of climate change in combination with various human activities will influence environmental conditions and selective regimes for fish in the Baltic Sea during the coming century and onwards. It is therefore complicated to foresee which genetic makeups, spawning strategies, phenotypic trait value combinations, and behaviors that will be most successful in the future. Consequently, it is essential that whatever management actions are taken, they must be planned and designed to ensure that genetic variation and phenotypic diversity (e.g., with regard to seasonal timing of reproduction, place of spawning, growth trajectories, size and age at maturity, and reproductive allocation strategies) are maintained, both among populations and among individuals within populations (Wennersten and Forsman 2012). Thus, if pike are to be translocated and released to founder new sub-populations, individuals used for the founder group(s) should be selected such that they are as different and variable as possible, because greater founder diversity generally promotes establishment success and reduces extinction risk (Forsman 2014). Greater genetic and phenotypic variation also enables populations to cope with environmental change and adapt to novel conditions by means of evolutionary modifications.

It should be emphasized here that benefits of increased variation apply with certainty only insofar as the variation is based upon individuals that originate from the same source population. The consequences of interbreeding between individuals from different populations can be positive, negative, or neutral depending on the evolutionary history and properties of the populations involved (McClelland and Naish 2007b; Rius and Darling 2014). Management actions should therefore rely primarily on methods and approaches that do not involve the mixing of individuals and genotypes that originate from different sub-populations. In the case of pike in the Baltic Sea, we offer two suggestions that comply with this advice: (i) productivity of extant freshwater spawning populations can be boosted by continuing to modify wetlands such that they promote spawning success and recruitment of pike; and (ii) new sub-populations of brackish water spawning pike populations can potentially be created by transferring fry and imprinting them on currently deserted but seemingly suitable spawning environments.
